# Evaluating the Acceptability and Appropriateness of the Augmented Reality Home Assessment Tool (ARHAT): Qualitative Descriptive Study

**DOI:** 10.2196/44525

**Published:** 2023-09-27

**Authors:** Beth Fields, McKenzie Fitzpatrick, Lauryn Kinney, Jenny Lee, Bryce Sprecher, Ross Tredinnick, Kevin Ponto, Jung-hye Shin

**Affiliations:** 1 Department of Kinesiology, University of Wisconsin Madison, Madison, WI, United States; 2 Department of Design Studies, University of Wisconsin Madison, Madison, WI, United States; 3 Wisconsin Institute for Discovery, University of Wisconsin Madison, Madison, WI, United States

**Keywords:** technology, aging in place, augmented reality, home modification, mobile, assessment, mobile application, qualitative study, environmental barrier

## Abstract

**Background:**

The Augmented Reality Home Assessment Tool (ARHAT) is a mobile app developed to provide rapid, highly accurate assessments of the home environment. It uses 3D-capture technologies to help people identify and address functional limitations and environmental barriers.

**Objective:**

This study was conducted to gain stakeholder feedback on the acceptability and appropriateness of the ARHAT for identifying and addressing barriers within home environments.

**Methods:**

A qualitative descriptive study was conducted because it allows for variability when obtaining data and seeks to understand stakeholders’ insights on an understudied phenomenon. Each stakeholder group (occupational therapists, housing professionals, and aging adult and caregiver “dyads”) participated in a 60-minute, web-based focus group via a secure Zoom platform. Focus group data were analyzed by 2 trained qualitative research team members using a framework method for analysis.

**Results:**

A total of 19 stakeholders, aged from 18 to 85+ years, were included in the study. Of the occupational therapists (n=5, 26%), housing professionals (n=3, 16%), and dyads (n=11, 58%), a total of 32% (n=6) were male and 68% (n=13) were female, with most living in the Midwestern United States (n=10, 53%). The focus group data demonstrate the acceptability and appropriateness of the workflow, style, measurement tools, and impact of the ARHAT. All stakeholders stated that they could see the ARHAT being used at many different levels and by any population. Dyads specifically mentioned that the ARHAT would allow them to do forward planning and made them think of home modifications in a new light.

**Conclusions:**

Stakeholders found the ARHAT to be acceptable and appropriate for identifying and addressing functional limitations and barriers in the home environment. This study highlights the importance of considering the workflow, style, measurement tools, and potential impact of home assessment technology early in the developmental process.

## Introduction

Nearly 90% of adults wish to age independently or remain in their home and community as long as possible [1,2]. Yet as people age, they are more likely to experience physical and cognitive declines and symptoms associated with chronic health conditions, which result in functional impairments that can impact their safety and ability to age independently [3]. Evidence demonstrates that addressing aging adults’ functional limitations in their home environment enables people to continue living at home instead of moving into an institutional care facility [4]. However, there are relatively few standardized mobile apps on the market that help people identify and address functional limitations and barriers in the home environment [5,6]. This is problematic because environmental barriers increase the risks of functional limitations and reduce the level of independence one has when choosing to age in place safely.

The Augmented Reality Home Assessment Tool (ARHAT) is a new tool aiming to address these problems. This mobile app helps people identify and address functional limitations and environmental barriers in their home and environments. In comparison to other mobile apps, the ARHAT is guided by the Housing Enabler (HE) [7] and the 2010 American Disability Act (ADA) [8] and provides rapid, highly accurate assessments of home environments by using 3D-capture technologies. The HE provides equal emphasis on the functional limitations of the individual and housing configurations based on the Ecological Theory of Aging and its foundational concept of the Person-Environment fit [9]. The HE uses 14 different types of functional limitations, focusing on the symptoms rather than a diagnosis of disease. Additionally, the ADA provides guidelines to ensure all spaces promote accessibility and usability for individuals with disabilities [8]. Together, the HE and ADA provide a solid foundation for guiding the ARHAT’s approach to assessment.

**Figure 1.**
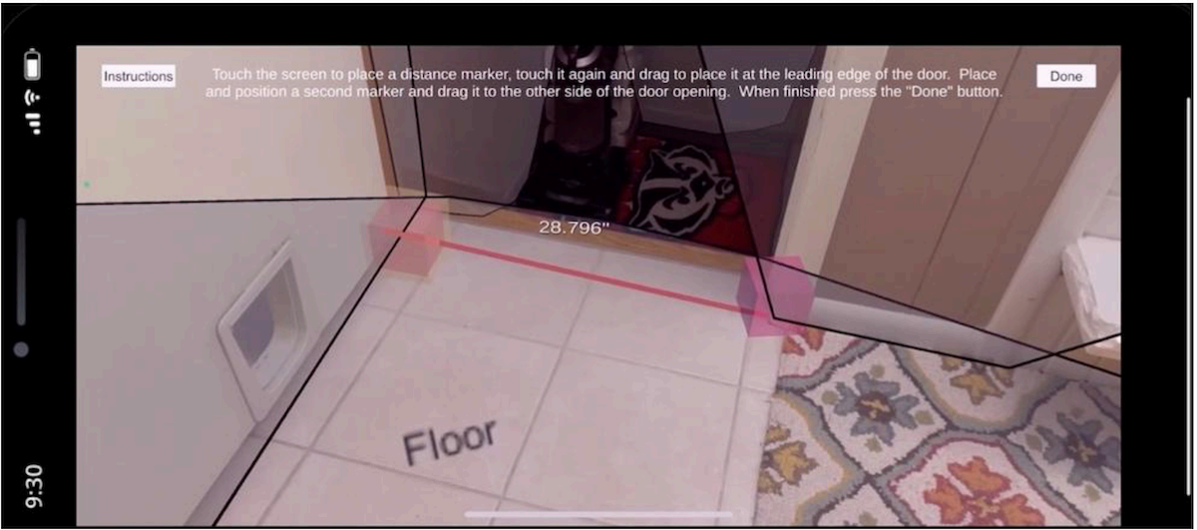
Distance tool.

The ARHAT is comprised of 3 unique approaches to assess the home environment: by selecting either a functional limitation (eg, vision impairment), a space (eg, the bathroom), or the whole house. Through each approach, users are prompted to learn about and use many augmented reality (AR) measurement tools, including tools for measuring distance, incline, illumination, radius, and maneuverable space. As individuals use these measurement tools in the ARHAT, they are provided information on whether their home is compliant with the ADA guidelines. Distance measurements are performed when the user touches areas on the screen they would like to measure. Two or more end points can be adjusted to measure both vertical and horizontal distances, for example, as shown in Figure 1 when performing a doorway measurement.

**Figure 2.**
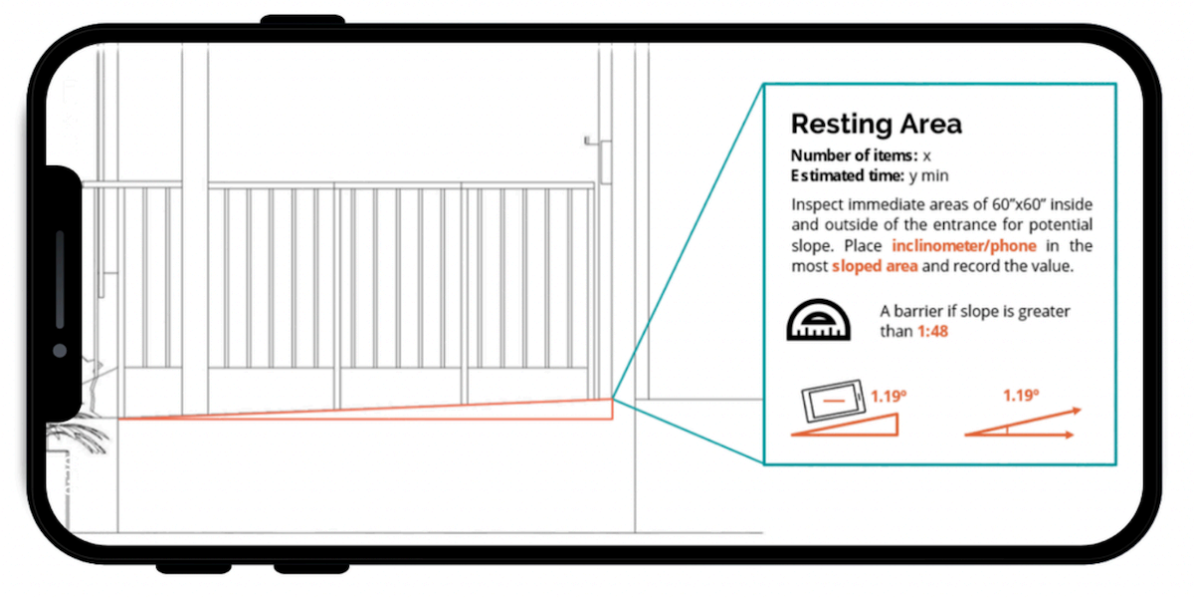
Incline tool.

Resting areas are traditionally measured using inclinometers to assess the slope of a resting area for accessibility. The ARHAT uses sensors inside the phone to measure slope when the phone is set on the ground (Figure 2).

**Figure 3.**
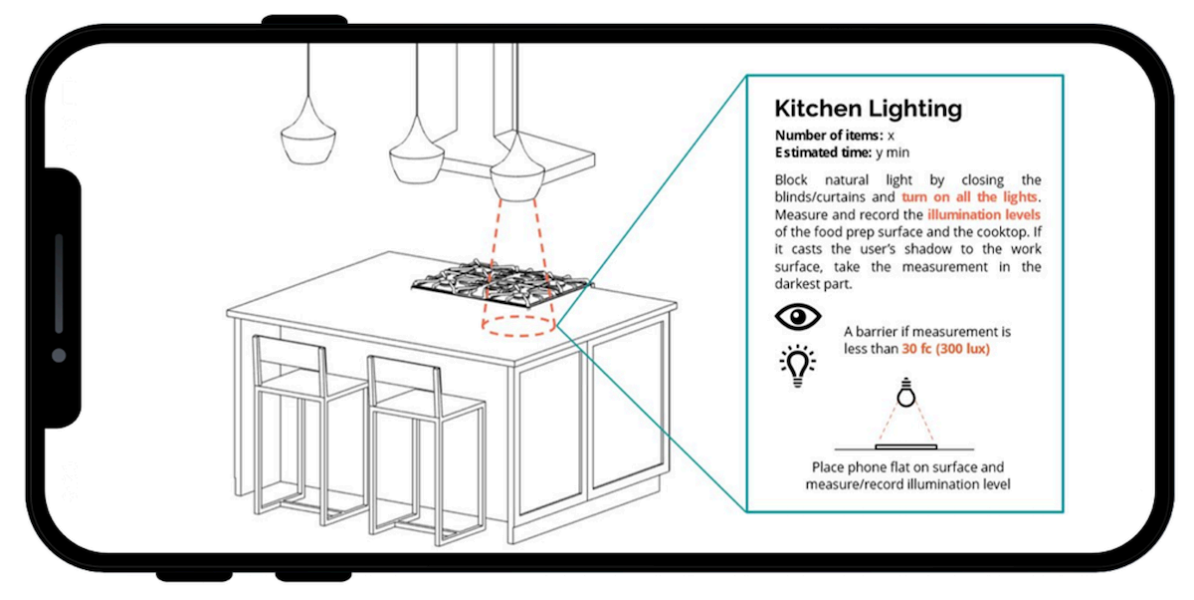
Illumination tool.

The ARHAT adds an objective way to measure whether illumination is sufficient for accessibility in an area; otherwise, it may become a safety concern. The ARHAT uses light sensors embedded in the phone to perform a similar assessment when placed on critical work surfaces (Figure 3).

**Figure 4.**
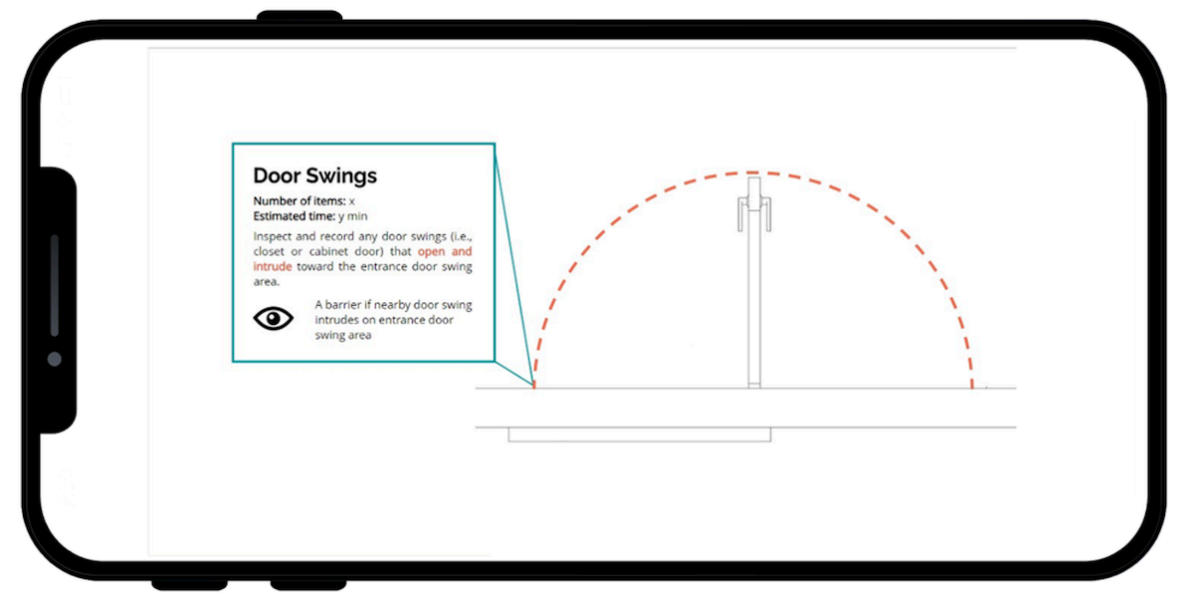
Radius tool.

The radius tool allows for direct visualization of the radius of a door swing, for example, to aid in the visual inspection of obstructions (Figure 4).

**Figure 5.**
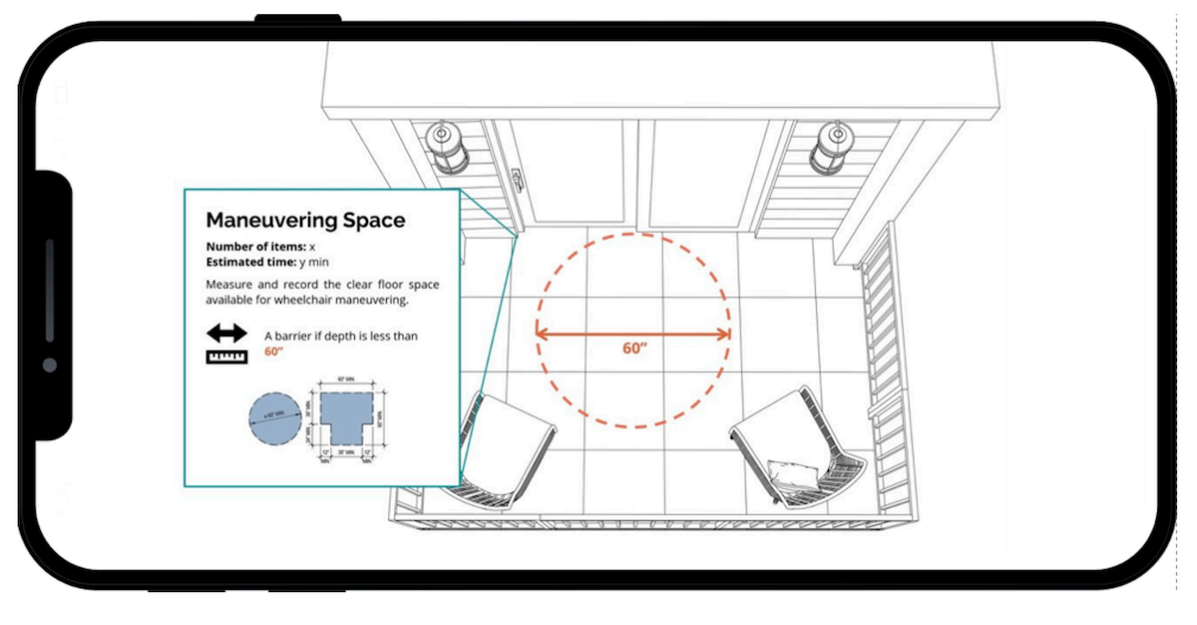
Maneuvering visualization tool.

Lastly, the maneuvering visualization tool allows the user to see a colored area on the floor that represents the measured maneuvering area for a wheelchair user (Figure 5).

Despite the promising utility for the ARHAT, the appropriateness and acceptability of the tool must be examined before implementation in the home environment. Appropriateness is defined as “the perceived fit, relevance, or compatibility of the innovation to address a current issue,” and acceptability is defined as “the perception among implementation stakeholders that a given treatment, service, practice, or innovation is agreeable, palatable, or satisfactory” [10]. These constructs are important to consider early in the ARHAT development process, as they serve as indicators of implementation success. Therefore, the purpose of this study was to assess the appropriateness and acceptability of the ARHAT. As a result of this study, the ever-growing number of people wanting to safely age in place will have a mobile app to help them identify and address barriers within their home environment.

## Methods

### Design

A qualitative descriptive study [11] was conducted. This design was selected because it allows for variability when obtaining data and seeks to understand stakeholders’ insights on an understudied phenomenon [11].

### Stakeholders

We purposely recruited occupational therapists (OTs), housing professionals (HPs), and aging adult and caregiver (“family members or friends”) dyads through social media sites and email listservs, including Twitter, Facebook, caregiving and aging networks (ie, Center for Aging Research and Education), and national organization community boards (ie, American Occupational Therapy’s Productive Aging Group). These 3 stakeholder groups were targeted because of their expertise in home assessments, home modifications, functional limitations, and aging in place. Inclusion criteria for OTs were having had 5 years of professional experience in their respective positions and speaking English. Inclusion criteria for HPs were having had 5 years of professional experience in their respective positions, having earned the Certified Aging-In-Place Specialist designation, and speaking English. Inclusion criteria for aging adults were being at least 60 years or older, speaking English, and living at home. Inclusion criteria for caregivers were being at least 18 years of age and providing unpaid care to a relative or partner aged 60 years or older to assist them in taking care of themselves. No stakeholders were excluded based on their sex, gender, race, ethnicity, or socioeconomic status.

From the recruitment materials, stakeholders interested in the study were directed to either email or call the research team. Research team members then followed up with interested stakeholders to share more study details, go over consent, and confirm next steps. All stakeholders were informed that the study was completely voluntary and that they could withdraw at any time.

### Ethical Considerations

Based on the study procedures, the Institutional Review Board at the University of Wisconsin-Madison (#2021-0856) determined this research to be no more than minimal risk and qualified as exempt from ethics approval. For these reasons, verbal consent was received from all stakeholders in this study.

### Data Collection

Each stakeholder group participated in a 60-minute, web-based focus group via a secure Zoom platform (Zoom Video Communications). Stakeholders were emailed a Zoom link, an agenda, and a demographic survey 1 week prior to the focus group. Demographics that were collected included gender, education, geographic location, age, occupation, and race and ethnicity. Each focus group was led by a trained qualitative research member (BF). Two other members of the research team (LK and MF) were responsible for taking notes during each focus group. During each focus group, stakeholders first introduced themselves. They then viewed a demonstration video of the ARHAT that walked them through the use of the AR technologies. After the demonstration video, the stakeholders were asked open-ended questions to learn their perspectives on the ARHAT’s acceptability and appropriateness [10]. For example, stakeholders were asked how they could see the ARHAT being used within their occupation or which color scheme was preferred. See Multimedia Appendix 1 for the focus group guides. All focus groups were recorded, transcribed, and checked for accuracy prior to data analysis.

### Data Analysis

Focus group data were analyzed by 2 trained qualitative research team members (LK and MF). The research team selected a framework method for analysis [12] because coding was guided by the overall organization of the focus groups. Specifically, the team members completed the following three steps: (1) read transcribed interviews, (2) applied existing categories to the data using the focus group guides, and (3) condensed codes into themes. All stakeholder interviews were transcribed and coded using NVivo 12 Pro (Lumivero). To ensure the reliability of coding, research team members performed a reliability check by comparing coding in a one-on-one meeting. Data were then summarized and discussed with the research team (LK, MF, BF, JHS, KP, RT, BS, and JL) to understand the strengths and areas for improvement of the ARHAT.

## Results

### Stakeholder Demographics

A total of 19 stakeholders, aged from 18 to 85+ years, were included in the study. Of the OTs (n=5, 26%), HPs (n=3, 16%), and dyads (n=11, 58%), a total of 32% (n=6) were male and 68% (n=13) were female, with most living in the Midwestern United States (n=10, 53%). Other stakeholders resided in the Northeastern (n=4, 21%), Western (n=1, 4%), Southeastern (n=2, 11%), and Southwestern (n=2, 11%) United States. Stakeholders were predominantly White (n=17, 89%), with 1 (5%) American Indian or Alaska Native and 1 (5%) African American stakeholder. The education of the 19 stakeholders varied, as 16% (n=3) held a doctorate-level degree, 26% (n=5) had obtained their master’s degree, and the majority (n=18, 94%) reported attending some college.

Data demonstrate the acceptability and appropriateness of the ARHAT and are organized around the focus group guides: workflow, style, measurement tools, and impact. To illustrate the acceptability and appropriateness of the ARHAT, direct quotes are provided for each stakeholder group.

### Workflow

To increase workflow usability, all stakeholder groups stated that they would like to see more prompting and call-out boxes within the ARHAT for further clarification. Moreover, during each focus group, stakeholders sparked questions about what the output data would look like from the ARHAT. They also inquired about how they would be able to use the information collected through the mobile app. Stakeholders recommended inserting a screen at the beginning of the ARHAT to describe how the data could be used, a preview of what the data would look like after measurements were taken, and how to export the data after completing an assessment. Demonstration videos within the ARHAT were suggested, as stakeholders thought this would be useful to all users. Dyad 1 stated that “I think it would be helpful if you’re going to do a measurement you could have an option to click a quick tutorial…then you know exactly what to do and then you do it yourself.” Stakeholders specifically mentioned a drop-down screen with a list of video tutorials would be the easiest to navigate, allowing users to stay within the ARHAT and not seek out resources outside of the mobile app. Overall, all stakeholder groups appreciated the simple to complex design and thought that it would be user-friendly for people of any age. For example, HP1 stated that “it’s pretty straightforward and seems easy to use with a swipe.”

### Style

**Figure 6.**
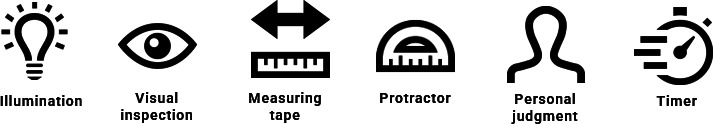
Measurement icons.

All stakeholder groups provided feedback on the ARHAT’s use of font, icons, and color. The font pairing throughout the ARHAT consists of Roboto Bold and Open Sans. In addition, 6 measurement icons are used throughout the ARHAT that guides the assessment process. These 6 icons are illumination, visual inspection, measuring tape, protractor, expert judgment, and timer (Figure 6).

Dyads reported that the font size was too small throughout the ARHAT, especially for a user that may have a vision impairment. Dyad 2 stated that “on any given screen or slide, the font looks very little.” Additionally, the bold font used throughout was difficult for some stakeholders to read. An area of confusion across all focus groups was the “expert judgment” icon. All stakeholders needed clarification on whether this icon was supposed to represent the user using their judgment or if it was prompting the user to seek out an expert opinion. OTs recommended that the ARHAT could use “personal judgment” as the title for this icon to avoid confusion. HPs also mentioned how enabling a 2-finger zoom-in feature on the app would be beneficial for users of the ARHAT. They recognized that this feature is allowed in some apps and thought that this could be useful for small font sizes or images throughout the ARHAT.

**Figure 7.**
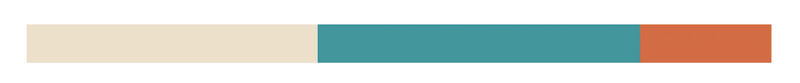
Color palette.

Stakeholders were shown a colored version of the ARHAT, along with a black-and-white version. Each stakeholder group was fond of the use of color throughout the ARHAT, as long as a specific color was not implying importance. OT1 stated that “I don’t know if color sometimes alarms you to something that you may think this is more important for me to assess.” All stakeholders were able to view 3 different color palettes, and collectively, they all agreed that the color palette consisting of teal, tan, and orange was the easiest to see throughout (Figure 7).

Dyad 3 stated that “I think this is a good example of using color to illustrate exactly what you’re trying to get to in a very quick way,” and D4 stated that “I think contrast is important.” Additionally, all stakeholder groups found all the functional limitation icons to be easily recognizable and, overall, liked the integration of the functional limitation icons throughout the ARHAT.

### Measurement Tools

One limitation that was discussed by OTs and dyads was that all users might not be able to perform all the measurements within the ARHAT independently. For example, some OTs shared that aging adults may not be able to bend over to place their phone on the ground to measure the slope of a ramp. Dyad 3 further clarified that someone with a “physical limitation” may not be able to “get down on their knees to put the camera on the porch just to see the angle.” Additionally, the distance tool used to measure the width of doors and entryways was critiqued, as 3D blocks (as seen in Figure 1) are used to line up this measurement. All stakeholder groups thought that the 3D blocks might be challenging for users because the 3D blocks may be difficult to align to get an accurate measurement. Instead of using 3D blocks, HPs recommended pinpoints, as they thought that it would be easier to get an accurate measurement by placing the points on specific areas. OTs critiqued the call-out boxes that are used within each measurement tool and suggested incorporating the ADA guidelines into the call-out boxes. They also mentioned the possibility that users of the ARHAT may not be familiar with the ADA, and it was suggested that explaining the guidelines somewhere within the tool could be helpful for further clarification for all users. Lastly, all stakeholder groups liked the lumen measurement but thought that the correct amount of light is different for everyone, especially those with visual impairments. OT3 stated that “people’s vision skills, vision abilities, are so variable.” Dyads were also curious if any settings on the user’s mobile device would need to be enabled before using the app to access the ARHAT, or if all capabilities needed for the measurement tool would be automatically turned on.

### Impact

All stakeholder groups stated that they could see the ARHAT being used at many different levels and by any population. Dyads specifically mentioned that the ARHAT would allow them to do forward planning and made them think of home modifications in a new light. OTs suggested that the ARHAT would be a great resource for people living in rural areas with limited resources. OT2 stated that “our county it’s very rural, it’s very sparsely populated, it’s a long drive to a healthcare facility or for the practitioner to go to the home.” The ARHAT could provide those with limited access to health care services the opportunity to complete in-home assessments, such as those conducted by OTs. The ARHAT could also be beneficial for individuals who are discharged from medical care and need to make their homes accessible but do not know where to start. OT3 stated that “rehab centers being able to say you can get this app and figure out if you think mom’s wheelchair will work in your house” is just one example of how the ARHAT may impact patient discharge planning.

OTs enjoyed the possibilities of this tool, as it would allow for a quicker evaluation and measurement process, as well as allowing them to record their measurements within a mobile app rather than on paper. They liked the ability to show clients a tool such as this and then have clients report back their information to provide modification suggestions without having to go into the home. The HPs identified the measurement tools as a beneficial aspect of the ARHAT. They stated that these tools would allow for easy assessment within the home. Dyads enjoyed the overall style of the tool, noting the clear layout and flow of the ARHAT. Dyad 10 shared that “I think it would be pretty easy for aging adults to use, even those who may not be familiar with AR technology.”

## Discussion

### Principal Findings

This study was conducted to gain stakeholder feedback on the acceptability and appropriateness of the ARHAT for identifying and addressing barriers within home environments. Three main findings were revealed from the stakeholders in this study: (1) more guidance is needed on how to use data collected from the ARHAT and ADA guidelines, (2) additional resources such as videos demonstrating the AR tools would increase the usability of the ARHAT across age groups, and (3) the ARHAT is an acceptable and appropriate tool for helping people safely age in place. The research team is using these findings to revise the ARHAT and improve future usability and adoption.

The ARHAT has the potential to provide individuals with an easy-to-use tool for assessing the home and identifying barriers in the environment. The ADA guidelines are a major component to the app development and key to understanding the data the ARHAT collects. We found that users of the ARHAT may benefit from learning the ADA guidelines directly through the mobile app itself. The ADA guidelines were developed to protect individuals who have one or more “major life activities” (eg, caring for oneself, walking, lifting, and bending) limited due to a physical or mental impairment [13]. The ARHAT would aid in the home assessment process of those seeking to age in place while managing their major life activities. Furthermore, we found that users may also benefit from knowing what the output data will look like before beginning an assessment and how they are able to disperse this information to potential home contractors, OTs, or family members.

Since AR is relatively new and constantly evolving, many people, especially older adults, may not be familiar with the technology or feel confident when using it for a home assessment. For example, intelligent display technology, 3D registration technology, and intelligent interaction technology are all versions of AR that allow for a magnitude of benefits for various applications [14]. One potential solution to account for differentiating skill levels in the realms of technology is to embed demonstration videos within the mobile app itself. OTs, HPs, and dyads all emphasized the impact that including demonstration videos would have on making the ARHAT user-friendly across all populations. According to Seifert and Schlomann [15], “developers, practitioners, and researchers in the field must acknowledge digital inequalities and provide older adults with training tools, support services, and digital solutions that consider their heterogeneous backgrounds and needs.” The addition of resources within the tool may also be less time-consuming for users, as information would be readily available to them. This would also aid in mitigating the limited knowledge of AR for some populations who are less familiar with technology [16].

According to Ahn et al [2], “prior research on gerontology and housing has frequently adopted a perspective that aging in place is the goal,” yet hiring a professional to conduct a home assessment to support aging in place can be time-consuming, expensive, or out of one’s comfort zone [17]. The ARHAT is an innovative tool that can provide people the opportunity to conduct their own home assessment, eliminating the need to hire or bring a professional in. As there is a growing acceptance of technology among the aging population, the ARHAT can help alleviate the common concerns of aging in place by providing further insight on whether their homes are compliant with the ADA guidelines [18]. Frequently, aging adults are unaware of their housing limitations [17]. The AR measurement tools embedded in the ARHAT will allow aging adults and others to identify limitations they may previously have not been aware of.

### Strengths and Limitations

There were many strengths to this study. Two trained qualitative research team members coded and analyzed data to verify the accuracy of the themes yielded from the focus groups. Additionally, purposeful sampling techniques were used to gain broad insight from diverse stakeholder groups. Expert knowledge from OTs, HPs, and aging adults and caregivers was gleaned to address potential challenges and solutions to increase the future usability and adoption of the ARHAT. All focus groups were held on the web, allowing stakeholders from across the United States to participate in this study. Furthermore, stakeholders who participated in this study were aged from 18 to 85+ years, contributing a wide range of personal experiences, knowledge, and perspectives to the findings.

This study is not without limitations. This study only included 19 stakeholders, with most stakeholders being female (n=13, 68%). The stakeholder group lacked diversity, as 89% (n=17) of the stakeholders were White. Furthermore, because of the web-based nature of the focus groups, the observation of nonverbal communication was difficult to obtain. Lastly, stakeholders were only shown a demonstration video of the ARHAT, which limits the research team’s ability to understand the usability features of the mobile app (eg, an older adult may have difficulty using the ARHAT due to their digital literacy level or deterioration of fine motor skills).

### Future Research

The research team is revising the ARHAT based on the focus group findings. Following these revisions, smartphones with the ARHAT installed will be disseminated to recruited stakeholders to gain feedback on their experience of using the mobile app in a real-world context. An electronic survey will be distributed to stakeholders to obtain their perceived usefulness and satisfaction of the ARHAT. They will also be able to provide insight on the workflow, style, and measurement tools through open-ended questions embedded in the survey. The team also recognizes that the goals and motivations for using the ARHAT may be different for each stakeholder group. A qualitative comparison analysis of focus group and survey data will be completed to better understand these potential differences.

### Conclusion

From the perspectives of the stakeholders in this study, the ARHAT is acceptable and appropriate for identifying and addressing functional limitations and barriers in the home environment. This study highlights the importance of considering the workflow, style, measurement tools, and potential impact of tools early in the developmental process. The feedback and areas for improvement received by a wide range of stakeholders involved in this study shed light on ways the ARHAT can reduce risk and improve the level of independence among people who wish to safely age in place.

## Supplementary material

10.2196/44525 Multimedia Appendix 1 Focus group guides.

## Abbreviations

ADA: American Disability Act
AR: augmented reality
ARHAT: Augmented Reality Home Assessment Tool
HE: Housing Enabler
HP: housing professional
OT: occupational therapist
