# Supplementary material for: Evaluating the Acceptability and Appropriateness of the Augmented Reality Home Assessment Tool (ARHAT): Qualitative Descriptive Study
Source: JMIR Aging. 2023 Sep 27;6:e44525. doi: 10.2196/44525 (PMC10547935; doi:10.2196/44525)
Supplement: Multimedia Appendix 1 [file aging-v6-e44525-s001.docx]

**Appendix**

**Augmented Reality Home Assessment Tool Focus Group Guide**

| **Title** | Augmented Reality Home Assessment Tool (ARHAT) |
| --- | --- |
| **Date** |  |
| **Name of Facilitator** |  |
| **Purpose of Focus Group** | We are developing an app that people can use to identify barriers in their home environment that may be impeding their ability to age in place. The purpose of this focus group is to receive feedback on our app. |
| **Target Group** | Occupational Therapists and Housing Professionals |

**Introduction**

I want to thank you all for taking the time to participate in our study; as a token of our appreciation for participating in the focus group today, you will be mailed a $75 stipend.

The purpose of today’s focus group is to first briefly present parts of the augmented reality home assessment app and then second, learn ideas that you may have to increase the usability of the app, as well any challenges you may expect to encounter while using the app. Information gained from this focus group will help us make improvements to the app before distributing to the general public.

Again, this focus group is part of a study at the University of Wisconsin-Madison. Your participation is voluntary. We are hoping to record this conversation, so that we can accurately represent everyone’s opinions and thoughts. We will de-identify all responses, so that they are not matched to you. The focus groups will be transcribed, coded, and summarized, and we will delete the recording after transcription. If you are comfortable with this conversation being recorded, please either unmute and say yes, or type yes in the chat now. If you are not comfortable with this conversation being recorded, we’d like to ask you to please excuse yourself from the Zoom focus group now.

(Wait a minute or two for responses, then press record on Zoom)

**Topic Guide and Questions**

**Background**

1. What qualifications or experiences do you have that would support the usability of this app?
2. What technology based assessments have been utilized by group members?
3. What home assessment tools/apps do you use?

**Input Seeking Based on the Presentation of the Initial Prototype**

1. Overall flow: Does the overall flow make sense and compatible with the way you normally work in the field?
2. UI/UX: Typefaces (readability, styles, sizes), layout (text hierarchy), is the style consistent?
3. AR measurement tool: Do you see yourself using these tools? What are possible challenges to using these tools?
4. Other graphical styles: Color choice (black & white vs. ull color), icons (does it make sense?), do the illustrations make sense (clarity), can you understand what the graphic is trying to communicate about the measurement?

**ARHAT General Impressions**

1. What were your initial impressions of the app that was presented today?
   1. Probe: target user, space v. disability type, icon clarity, instructions, etc.
2. Are there any suggestions to improve the content of the app that was presented today?
   1. Probe: If so, how?
3. Based on what you know now about the app, would it help fill a gap/need in the home assessment industry?
   1. Why or why not?
4. What resources would you expect to need to understand how to best utilize the app?
   1. For yourself?
   2. For Coworkers?
5. How do you see the app impacting your own practice, the home assessment industry, or larger health systems?
   1. Probe: Increase satisfaction, reduce health service utilization, increase safety/independence, etc.
6. What ADL’s do you feel this tool will aid in for age at home clients?
   1. IADL’s?
7. What populations do you envision utilizing the app most among your clients?

**ARHAT Challenges and Solutions**

1. Tell me about any challenges that you would expect to encounter with using this app.
   1. Probe: challenges across populations, home environments, devices/tech (e.g., Apple v. Android)
2. What potential solutions exist to address these challenges?

**Closing**

That brings us to the end of our focus group today. Again, thank you all so much for your time and participation. We value your feedback and look forward to making improvements to our app. If anyone has any additional questions or comments that they would like to share, please feel free to email me at X.

**Augmented Reality Home Assessment Tool Focus Group Guide**

| **Title** | Augmented Reality Home Assessment Tool (ARHAT) |
| --- | --- |
| **Date** |  |
| **Name of Facilitator** |  |
| **Purpose of Focus Group** | We are developing an app that people can use to identify barriers in their home environment that may be impeding their ability to age in place. The purpose of this focus group is to receive feedback on our app. |
| **Target Group** | Adult/Caregiver Participants |

**Introduction**

I want to thank you all for taking the time to participate in our study; as a token of our appreciation for participating in the focus group today, you will be mailed a $75 stipend.

The purpose of today’s focus group is to first briefly present parts of the augmented reality home assessment app and then second, learn ideas that you may have to increase the usability of the app, as well any challenges you may expect to encounter while using the app. Information gained from this focus group will help us make improvements to the app before distributing to the general public.

Again, this focus group is part of a study at the University of Wisconsin-Madison. Your participation is voluntary. We are hoping to record this conversation, so that we can accurately represent everyone’s opinions and thoughts. We will de-identify all responses, so that they are not matched to you. The focus groups will be transcribed, coded, and summarized, and we will delete the recording after transcription. If you are comfortable with this conversation being recorded, please either unmute and say yes, or type yes in the chat now. If you are not comfortable with this conversation being recorded, we’d like to ask you to please excuse yourself from the Zoom focus group now.

(Wait a minute or two for responses, then press record on Zoom)

**Topic Guide and Questions**

**Background**

1. What qualifications or experiences do you have that would support the usability of this app?
2. What technology-based assessments have been utilized by group members?
3. How do you think our app compares to other home assessments on the market?

**Input Seeking Based on the Presentation of the Initial Prototype**

1. Overall flow: Does the overall flow make sense and compatible with the way you normally work in the field?
2. UI/UX: Typefaces (readability, styles, sizes), layout (text hierarchy), is the style consistent?
3. AR measurement tool: Do you see yourself using these tools? What are possible challenges to using these tools?
4. Other graphical styles: Color choice (black and white vs. full color), icons (does it make sense?), do the illustrations make sense (clarity), can you understand what the graphic is trying to communicate about the measurement?

**ARHAT General Impressions**

1. What were your initial impressions of the app that was presented today?
   1. Probe: target user, space v. disability type, icon clarity, instructions, etc.
2. Are there any suggestions to improve the content of the app that was presented today?
   1. Probe: If so, how?
3. Based on what you know now about the app, would it help fill a gap/need in the home assessment industry?
   1. Why or why not?
4. What resources would you expect to need to understand how to utilize the app?
5. How do you think the app could impact older adults with disabilities?
   1. Family /Caregivers?

**ARHAT Challenges and Solutions**

1. Tell me about any challenges that you would expect to encounter with using this app.
   1. Probe: challenges across populations, home environments, devices/tech (e.g., Apple v. Android)
2. What potential solutions exist to address these challenges?

**Closing**

That brings us to the end of our focus group today. Again, thank you all so much for your time and participation. We value your feedback and look forward to making improvements to our app. If anyone has any additional questions or comments that they would like to share, please feel free to email me at X.
